# Screening and structure-based modeling of T-cell epitopes of Nipah virus proteome: an immunoinformatic approach for designing peptide-based vaccine

**DOI:** 10.1007/s13205-015-0303-8

**Published:** 2015-05-19

**Authors:** Mohit Kamthania, D. K. Sharma

**Affiliations:** Mangalayatan University, Aligarh-Mathura Highway, Beswan, Aligarh, Uttar Pradesh India; Jiwaji University, Gwalior, Madhya Pradesh India; Department of Zoology, Government Post Graduate College, Guna, Madhya Pradesh India

**Keywords:** Nipah virus, Molecular modeling, T-cell epitope, Vaccine designing, MHC class I alleles

## Abstract

Identification of Nipah virus (NiV) T-cell-specific antigen is urgently needed for appropriate diagnostic and vaccination. In the present study, prediction and modeling of T-cell epitopes of Nipah virus antigenic proteins nucleocapsid, phosphoprotein, matrix, fusion, glycoprotein, L protein, W protein, V protein and C protein followed by the binding simulation studies of predicted highest binding scorers with their corresponding MHC class I alleles were done. Immunoinformatic tool ProPred1 was used to predict the promiscuous MHC class I epitopes of viral antigenic proteins. The molecular modelings of the epitopes were done by PEPstr server. And alleles structure were predicted by MODELLER 9.10. Molecular dynamics (MD) simulation studies were performed through the NAMD graphical user interface embedded in visual molecular dynamics. Epitopes VPATNSPEL, NPTAVPFTL and LLFVFGPNL of Nucleocapsid, V protein and Fusion protein have considerable binding energy and score with HLA-B7, HLA-B*2705 and HLA-A2MHC class I allele, respectively. These three predicted peptides are highly potential to induce T-cell-mediated immune response and are expected to be useful in designing epitope-based vaccines against Nipah virus after further testing by wet laboratory studies.

## Introduction

Nipah virus (NiV) was isolated in 1999 and was identified as the etiological agent responsible for an outbreak of severe respiratory disease and fatal encephalitis in Malaysia and Singapore in pigs and humans (Chua et al. 1999). During the first NiV outbreak, the virus infected both pigs and humans, in addition to a small number of cats, dogs and horses (Chua et al. 2000; Epstein et al. 2006). NiV, a member of the family Paramyxoviridae, possesses a negative-sense, non-segmented RNA genome that is 18,246 nt (Malaysian isolate) or 18,252 nt (Bangladesh isolate) in length (Harcourt et al. 2005). It has six transcription units that encode six structural proteins, the nucleocapsid (N), phosphoprotein (P), matrix protein (M), fusion protein (F), glycoprotein (G) and polymerase (L). Similar to other paramyxoviruses, the P gene of NiV expresses four proteins, namely P, V, W and C (Harcourt et al. 2000; Wang et al. 2001).

In Bangladesh, 135 probable or confirmed cases of NiV infection in humans were identified from 2001 through 2008; 98 (73 %) were fatal (Luby et al. 2009). Active Nipah virus encephalitis surveillance identified an encephalitis cluster and sporadic cases in Faridpur, Bangladesh, in January 2010. 16 case patients were identified, in which 14 of these patients died (Sazzad et al. 2013).

Vaccination is the most effective of all the medical interventions to save human and animal lives and to increase production (Horzinek 1999; Tang et al. 2012a). Compared to the conventional vaccines, peptide- or epitope-based vaccines are easy to produce, more specific, cost effective, less time consuming and also safe (Kumar et al. 2013). It is well established that T cells play a critical role in inducing cellular immune response against foreign antigens but they recognize antigenic fragments only when they are associated with major histocompatibility complex (MHC) molecules exposed on surface of all vertebrate cells (Shekhar et al. 2012; Mohabatkar and Mohammadzadegan 2007). Immunoinformatics approach uses computational algorithms to predict potential vaccine candidates or T-cell epitopes. The advantage of a peptide- or epitope-based vaccine is the ability to deliver high doses of the potential immunogen and at a low cost (Von Hoff et al. 2005; Tang et al. 2012b). Viral protein which could act as a vaccine candidate must be surface-exposed, antigenic and responsible for pathogenicity (Cerdino-Tarraga et al. 2003; Verma et al. 2011).

## Materials and methods

The amino acid sequence of Nucleocapsid, phosphoprotein, matrix, fusion, glycoprotein, L protein, W protein, V protein and C protein was retrieved from the protein sequence database of NCBI (http://www.ncbi.nlm.nih.gov/protein) and their accession number is shown in Table 1.

**Table 1:** Scores generated by ProPred for MHC class I

Protein	Accession no.	Length of amino acid	Start position	Peptides/epitope	Allele	ProPred score
Nucleocapsid	ACT32611	532	38	VPATNSPEL	HLA-B7	1200
			474	SLLNLRSRL	HLA-A2	5968.882
Phosphoprotein	ACT32612	709	624	EPYGAAVQL	HLA-B*5102	2640
			464	NPADDNDSL	HLA-B*5102	550
Matrix	ACT32613	352	201	IAFNLLVYL	HLA-A*0201	4702.218
			293	FQKNLCFSL	HLA-B*2705	3000
Fusion	ACT32614	546	209	LLFVFGPNL	HLA-A2	2678.131
			192	KQTELSLDL	HLA-B*2705	9000
			125	AQITAGVAL	HLA-B*2705	2000
Glycoprotein	ACT32615	602	247	RIIGVGEVL	HLA-B*2705	2000
			38	EGLLDSKIL	HLA-B*5102	4400
			45	ILSAFNTVI	HLA-A2	3901.211
L protein	ACT32616	2244	1688	NPQEKICVL	HLA-B*5102	2662
			1482	FPLWSTEEL	HLA-B*5102	5280
W protein	YP_007188592	449	66	DGDVERRNL	HLA-B*5101	520
V protein	NP_112023	456	186	NPTAVPFTL	HLA-B*2705	2000
			66	DGDVERRNL	HLA-B*5101	520
			316	KEEPPQKRL	HLA-B*2705	2000
C protein	NP_112024	166	116	PDMDLLQAL	HLA-B*2705	2000
			0	MMASILLTL	HLA-B*2705	2000

### Prediction of MHC class I binding peptides

The prediction of promiscuous MHC class I binding peptides was done using a popular immunoinformatic tool ProPred I (Singh and Raghava 2001). It is an online web tool which uses matrix-based method that allows the prediction of MHC-binding sites in an antigenic sequence for MHC class I alleles. It also allows the prediction of the standard proteasome and immunoproteasome cleavage sites in an antigenic sequence. The simultaneous prediction of MHC binders and proteasome cleavage sites in an antigenic sequence leads to the identification of potential T-cell epitopes.

### Structure-based modeling of T-cell epitopes

The PEPstr (peptide tertiary structure prediction server) server (Kaur et al. 2007) predicts the tertiary structure of small peptides with sequence length varying between 7 and 25 residues. The prediction strategy is based on the realization that β-turn is an important and consistent feature of small peptides in addition to regular structures. Thus, the methods use both the regular secondary structure information predicted from PSIPRED and β-turns information predicted from BetaTurns. The side-chain angles are placed using standard backbone-dependent rotamer library. The structure is further refined with energy minimization and molecular dynamic simulations using Amber version 6.

### Modeling and validation of MHC I alleles

The IMGT/HLA database (http://www.ebi.ac.uk/ipd/imgt/hla/intro.html) (Robinson et al. 2013) currently contains 10,103 allele sequences. In addition to the physical sequences, the database contains detailed information concerning the material from which the sequence was derived and data on the validation of the sequences. The IMGT/HLA database allows you to retrieve information upon a specific HLA allele (http://www.ebi.ac.uk/ipd/imgt/hla/allele.html) as named in the WHO Nomenclature Committee Reports. 3D structures of alleles were retrieved from IMGT/HLA database. Some of the allele’s structures, which are not presented in IMGT/HLA database, were modeled with the help of MODELLER 9.10. The stereochemical qualities of the alleles were checked by PROCHECK (Laskowski et al. 1993).

### Molecular docking

Docking of peptides and alleles structure was carried out using AutoDock 4.2 (Goodsell and Olson 1990; Morris et al. 1998). Gasteiger charges were added to the ligand and maximum six numbers of active torsion are given to the lead compound using AutoDock tool (http://autodock.scripps.edu/resources/adt). Kollaman charges and solvation term were added to the protein structure using AutoDock tool. The Grid for docking calculation was centered to cover the protein-binding site residues and accommodate ligand to move freely. During the docking procedure, a Lamarckian genetic algorithm (LGA) was used for flexible ligand rigid protein docking calculation. Docking parameters were as follows: 30 docking trials, population size of 150, maximum number of energy evaluation ranges of 250,000, maximum number of generations is 27,000, mutation rate of 0.02, cross-over rate of 0.8, other docking parameters were set to the software’s default values.

### Molecular dynamics simulation of epitope and HLA allele complex

Molecular dynamics simulation was done using the NAMD graphical interface module (James et al. 2005) incorporated visual molecular dynamics (VMD 1.9.2) (Humphrey et al. 1996). A protein structure file (psf) stores structural information of the protein, such as various types of bonding interactions. The psf was created from the initial pdb and topology files. The psfgen package of VMD is used to create this. To create a psf, we will first make a pgn file, which will be the target of psfgen. After running psfgen, two new files were generated protein pdb and protein psf and by accessing PSF and PDB files; NAMD generated the trajectory DCD file. Root mean square deviation (RMSD) of the complex was completed using rmsd tcl source file from the Tk console and finally rmsd dat was saved and accessed in Microsoft office excel 2007.

## Results and discussion

### Prediction and analysis of MHC class I binding peptides

The Nucleocapsid peptide VPATNSPEL at position 38–46 showed ProPred score of 1200 with HLA-B7 MHCI allele. The V protein peptide NPTAVPFTL at position 186–194 showed ProPred score of 2000 with the HLA-B*2705 allele. And the fusion protein peptide LLFVFGPNL at position 209–217 showed ProPred score of 2678.131 with the HLA-A2 allele. ProPred scores of peptides with MHC I alleles are shown in Table 1.

### Docking energy determination by AutoDock

3-D coordinate files of allele were obtained through IMGT/HLA database or model through MODELLER (Table 2) were validated using PROCHECK tool. After that, binding simulation studies show that nucleocapsid epitope VPATNSPEL with HLA-B7 allele, V protein epitope NPTAVPFTL with HLA-B*2705 allele as well as fusion epitope LLFVFGPNL with HLA-A2 allele formed stable HLA–peptide complexes with the energy minimization values of −5.07, −3.13 and −3.11 kcal/mol, respectively (Table 3). After docking studies, we determined the number of H bonds present in the stable complex formed.

**Table 2:** List of class I MHC alleles considered in this study for prediction of binding peptides

S. no.	Allele	Template (PDB ID)	Crystal structure/model
1	HLA-B7	3VCL	Crystal structure
2	HLA-A2	1AKJ	Crystal structure
3	HLA-B*5102	1E27	Model
4	HLA-A*0201	1AKJ	Crystal structure
5	HLA-B*2705	1HSA	Crystal structure
6	HLA-B*5101	1E27	Crystal structure

The table lists the PDB IDs of the template used for prediction and whether it was a crystal structure or a structural model

**Table 3:** Docking result of epitopes with allele structures

Protein	Peptide/epitope	Allele	BE	IME	IE	TorE	VdwE	EE
Nucleocapsid	VPATNSPEL	HLA-B7	−5.07	−12.52	−4.06	7.46	−9.47	−3.05
	SLLNLRSRL	HLA-A2	−1.45	−12.49	−4.94	11.04	−11.75	−0.74
Phosphoprotein	EPYGAAVQL	HLA-B*5102	−1.27	−9.33	−7.82	8.05	−9.57	0.24
	NPADDNDSL	HLA-B*5102	−0.65	−9.3	−0.89	8.65	−8.13	−1.16
Matrix	IAFNLLVYL	HLA-A*0201	−1.98	−11.52	−5.23	9.55	−11.3	−0.22
	FQKNLCFSL	HLA-B*2705	0.35	−10.39	−4.94	10.74	−8.36	−2.03
Fusion	LLFVFGPNL	HLA-A2	−3.11	−11.76	−4.98	8.65	−10.47	−1.29
	KQTELSLDL	HLA-B*2705	2.72	−8.32	−6.03	11.04	−8.14	−0.18
	AQITAGVAL	HLA-B*2705	−0.64	−8.39	−3.49	7.76	−6.6	−1.8
Glycoprotein	RIIGVGEVL	HLA-B*2705	0.36	−9.18	−3.76	9.55	−8.49	−0.69
	EGLLDSKIL	HLA-B*5102	0.05	−10.39	−5.75	10.44	−10.13	−0.26
	ILSAFNTVI	HLA-A2	−1.04	−9.98	−8.09	8.95	−9.94	−0.05
L protein	NPQEKICVL	HLA-B*5102	0.55	−10.49	−5.44	11.04	−10.62	0.14
	FPLWSTEEL	HLA-B*5102	1.31	−8.53	−6.14	9.84	−7.25	−1.28
W protein	DGDVERRNL	HLA-B*5101	2.58	−8.46	−3.91	11.04	−8.34	−0.12
V protein	NPTAVPFTL	HLA-B*2705	−3.13	−10.29	−3.5	7.16	−9.31	−0.98
	DGDVERRNL	HLA-B*5101	2.38	−8.66	−3.8	11.04	−7.75	−0.9
	KEEPPQKRL	HLA-B*2705	−0.26	−11.59	−4.3	11.34	−8.63	−2.97
C protein	PDMDLLQAL	HLA-B*2705	1.76	−8.09	−4.61	9.84	−6.07	−2.02
	MMASILLTL	HLA-B*2705	−2.38	−12.22	−2.54	9.84	−11.46	−0.76

*BE* binding energy, *IME* intermolecular energy, *IE* internal energy, *TorE* torsional energy, *VdwE* Vdw-lbDesolv energy, *EE* electrostatic energy

**Fig. 1:**
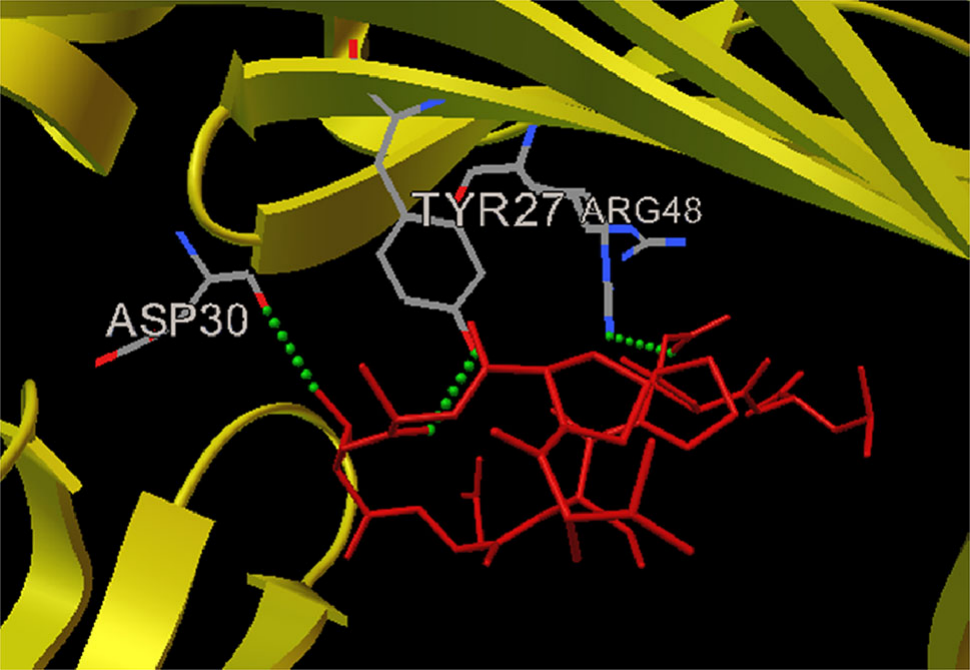
Docked nucleocapsid protein peptide VPATNSPEL-HLA-B7 allele complex depicting position of amino acids along with the formation of 3 H bonds with ASP30, TYR27 and ARG48

**Fig. 2:**
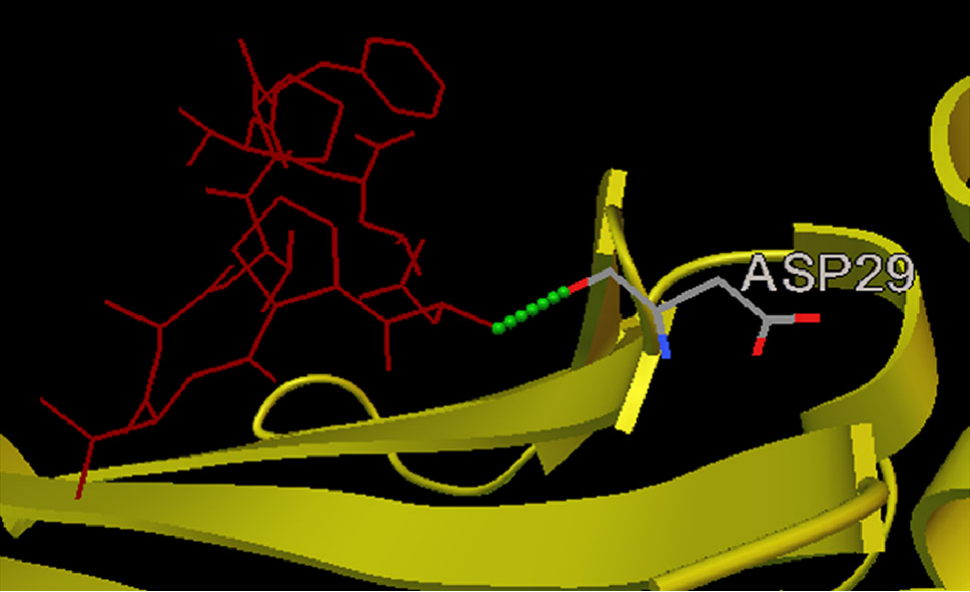
Docked V protein peptide NPTAVPFTL–HLA-B*2705 allele complex depicting position of amino acids along with the formation of 1 H bond with ASP29

**Fig. 3:**
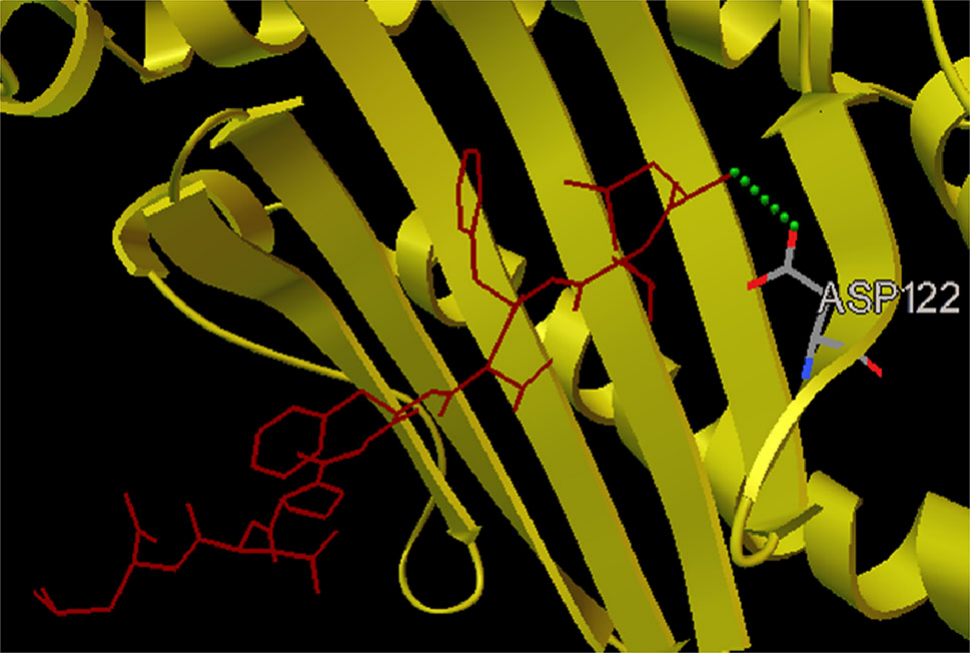
Docked fusion protein peptide LLFVFGPNL–HLA-A2 allele complex depicting position of amino acids along with the formation of 1 H bond with ASP122

Using AutoDock, it was found that three H-bonds were present in peptide VPATNSPEL-HLA-B7 allele complex as shown in (Fig. 1), first H-bond formed between allele residue ASP30:O with epitope amino acid THR4:OG1, second H-bond formed between allele residue ARG48:NH2 with epitope amino acid GLU8:OE1 and third H-bond formed between allele residue TYR27:OH with epitope amino acid ALA3:O, whereas one H-bond was present in V protein peptide NPTAVPFTL - HLA-B*2705 allele complex (Fig. 2) via residue ASP29:O with epitope amino acid ASN1:HN1. Fusion protein peptide LLFVFGPNL - HLA-A2 allele complex depicting position of amino acids along with formation of one H-bond with allele residue ASP122ASP122:OD2 with epitope amino acid LEU1:N (Fig. 3).

### Molecular dynamics simulation of peptide–allele complex through NAMD

**Fig. 4:**
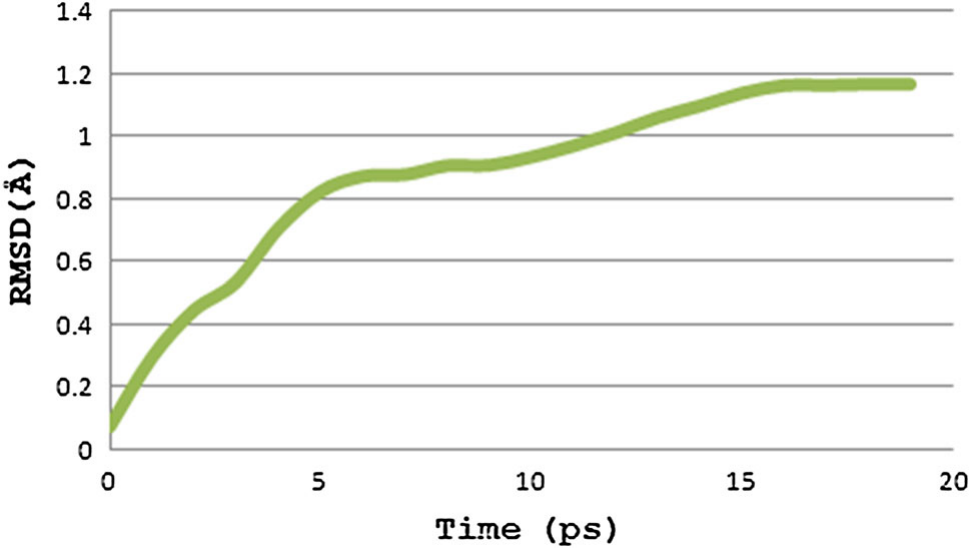
Graph displaying molecular dynamic simulation of Nucleocapsid peptide–allele complex, resulted in highest peak at 1.16 Å

**Fig. 5:**
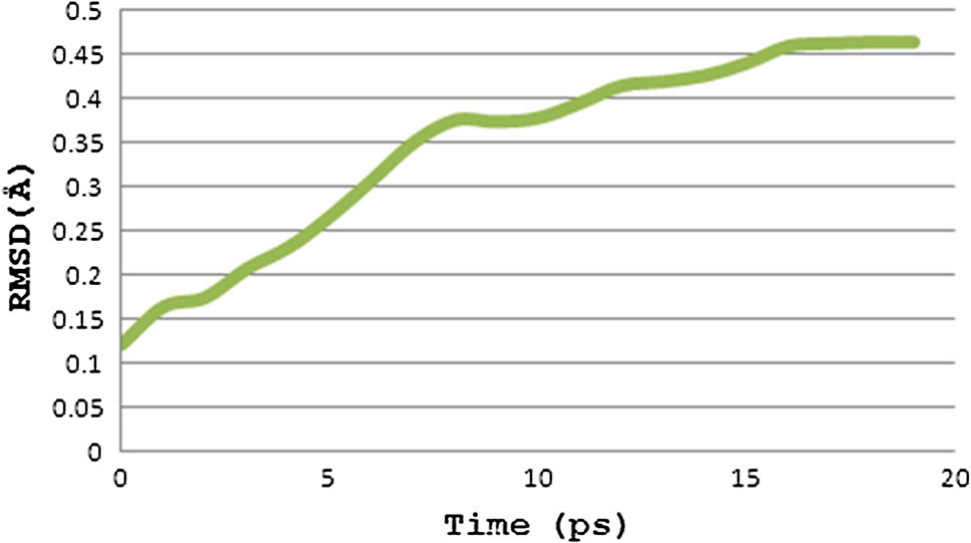
Graph displaying molecular dynamic simulation of V protein peptide–allele complex, resulted in highest peak at 0.46 Å

**Fig. 6:**
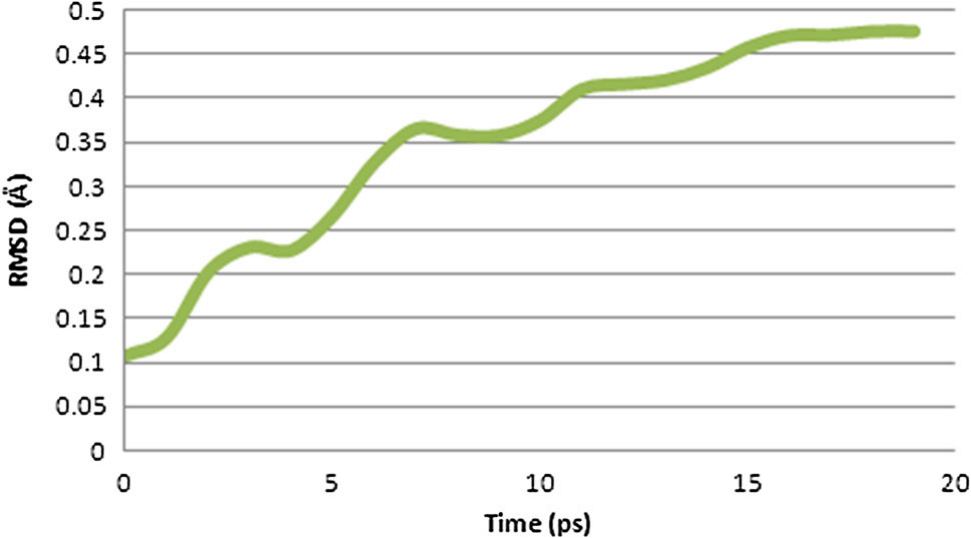
Graph displaying molecular dynamic simulation of fusion protein peptide–allele complex, resulted in highest peak at 0.47 Å

The peptide–allele complexes formed by AutoDock were subjected to molecular dynamics simulation and RMSD. Nucleocapsid epitope VPATNSPEL-HLA-B7 allele complex displayed the highest peak at RMSD value of 1.16 Å (Fig. 4). V protein peptide NPTAVPFTL-HLA-B*2705 allele complex resulted in highest peak at RMSD value of 0.46 Å (Fig. 5). And epitope LLFVFGPNL-HLA-A2 allele complex resulted in highest peak at RMSD value of 0.47 Å (Fig. 6).

Nipah virus (NiV) was associated with highly lethal febrile encephalitis in humans and a predominantly respiratory disease in pigs. Periodic deadly outbreaks, documentation of person-to-person transmission, and the potential of this virus as an agent of agroterror reinforce the need for effective means of therapy and prevention (Walpita et al. 2011). The current study incorporates immunoinformatics approach for reducing the time consumed in the long array of experiments to avoid hit and trial sets. Walpita et al. (2011) describe the vaccine potential of NiV proteins glycoprotein, fusion and matrix. Sakib et al. (2014) designed epitope-based peptides for the utility of vaccine development by targeting glycoprotein G and envelope protein F of Nipah virus (NiV) that, respectively, facilitates attachment and fusion of NiV with host cells. AutoDock resulted in good binding affinity along with H bonds at default parameters. The molecular dynamics simulation showed that complex formed between a peptide and allele was attaining proper stability by creating a parallelism in RMSD over a time window. The mentioned peptides can be either isolated or formulated for further in vitro and in vivo testings.

## Conclusion

The conclusion drawn from the present study is that the three epitopes VPATNSPEL, NPTAVPFTL and LLFVFGPNL of nucleocapsid, V protein and fusion protein, respectively, have considerable binding with HLA-B7, HLA-B*2705 and HLA-A2MHC class I allele and low-energy minimization values providing stability to the peptide–MHC complex. These peptide constructs may further be undergone wet laboratory studies for the development of targeted vaccine against Nipah virus.

## References

[CR1] Cerdino-Tarraga AM, Efstratiou A, Dover LG, Holden MTG, Pallen M (2003). The complete genome sequence and analysis of *Corynebacterium diphtheria* NCTC13129. Nucl Acids Res.

[CR2] Chua KB, Goh KJ, Wong KT, Kamarulzaman A, Tan PS, Ksiazek TG, Zaki SR, Paul G, Lam SK, Tan CT (1999). Fatal encephalitis due to Nipah virus among pig-farmers in Malaysia. Lancet.

[CR3] Chua KB, Bellini WJ, Rota PA, Harcourt BH, Tamin A (2000). Nipah virus: a recently emergent deadly paramyxovirus. Science.

[CR4] Epstein JH, Abdul Rahman S, Zambriski JA, Halpin K, Meehan G (2006). Feral cats and risk for Nipah virus transmission. Emerg Infect Dis.

[CR5] Goodsell DS, Olson AJ (1990). Automated docking of substrates to proteins by simulated annealing. Proteins.

[CR6] Harcourt BH, Tamin A, Ksiazek TG, Rollin PE, Anderson LJ (2000). Molecular characterization of Nipah virus, a newly emergent paramyxovirus. Virology.

[CR7] Harcourt BH, Lowe L, Tamin A, Liu X, Bankamp B (2005). Genetic characterization of Nipah virus, Bangladesh, 2004. Emerg Infect Dis.

[CR8] Horzinek MC (1999). Vaccination: a philosophical view. Adv Vet Med.

[CR9] Humphrey W, Dalke A, Schulten K (1996). VMD-visual molecular dynamics. J Mol Gr.

[CR10] James CP, Braun R, Wang W, Gumbart J, Tajkhorshid E, Villa E, Chipot C, Skeel RD, Kale L, Schulten K (2005). Scalable molecular dynamics with NAMD. J Comput Chem.

[CR11] Kaur H, Garg A, Raghava GPS (2007). PEPstr: a de novo method for tertiary structure prediction of small bioactive peptides. Protein PeptLett.

[CR12] Kumar A, Jain A, Shraddha Verma SK (2013). Screening and structure-based modeling of T-cell epitopes of Marburg virus NP, GP and VP40: an immunoinformatic approach for designing peptide-based vaccine. Trends Bioinform.

[CR13] Laskowski RA, MacArthur MW, Moss DS, Thornton JM (1993). PROCHECK—a program to check the stereochemical quality of protein structures. J App Cryst.

[CR14] Luby SP, Gurley ES, Hossain MJ (2009). Transmission of human infection with Nipah virus. Clin Infect Dis.

[CR15] Mohabatkar H, Mohammadzadegan R (2007). Computational comparison of T-cell epitopes of gp120 of Iranian HIV-1 with different subtypes of the virus. Pak J Biol Sci.

[CR16] Morris GM, Goodsell DS, Halliday RS, Huey R, Hart WE, Belew RK, Olson AJ (1998). Automated Docking using a Lamarckian genetic algorithm and empirical binding free energy function. J Comput Chem.

[CR17] Robinson J, Halliwell JA, McWilliam H, Lopez R, Parham P, Marsh SGE (2013). The IMGT/HLA Database. Nucleic Acids Res.

[CR18] Sakib MS, Md. Islam R, MahbubHasan AKM, NurunNabi AHM (2014) Prediction of epitope-based peptides for the utility of vaccine development from fusion and glycoprotein of Nipah virus using in silico approach. Adv Bioinform, Article ID 402492, p 17 10.1155/2014/402492 PMC4131549 25147564

[CR19] Sazzad HMS, Hossain MJ, Gurley ES, Ameen KMH, Parveen S, Islam MS (2013). Nipah virus infection outbreak with nosocomial and corpse-to-human transmission, Bangladesh. Emerg Infect Dis.

[CR20] Shekhar C, Dev K, Verma SK, Kumar A (2012). In-silico: screening and modeling of CTL binding epitopes of crimeancongo hemorrhagic fever virus. Trends Bioinform.

[CR21] Singh H, Raghava GPS (2001). ProPred: prediction of HLA-DR binding sites. Bioinformatics.

[CR22] Tang H, Liu XS, Fang YZ, Pan L, Zhang ZW (2012). The epitopes of foot and mouth disease. Asian J Anim Vet Adv.

[CR23] Tang H, Liu XS, Fang YZ, Pan L, Zhang ZW (2012). Advances in studies on vaccines of foot-and-mouth disease. Asian J Anim Vet Adv.

[CR24] Verma SK, Yadav SSP, Kumar A (2011). In silico T-cell antigenic determinants from proteome of H1N2 swine influenza A virus. Online J Bioinform.

[CR25] Von Hoff DD, Evans DB, Hruban RH (2005). Pancreatic cancer.

[CR26] Walpita P, Barr J, Sherman M, Basler CF, Wang L (2011). Vaccine potential of Nipah virus-like particles. PLoS One.

[CR27] Wang L, Harcourt BH, Yu M, Tamin A, Rota PA (2001). Molecular biology of Hendra and Nipah viruses. Microbes Infect.

